# Celiac Disease Is a Risk Factor for Mature T and NK Cell Lymphoma: A Mendelian Randomization Study

**DOI:** 10.3390/ijms24087216

**Published:** 2023-04-13

**Authors:** Rafael Martín-Masot, Marta Herrador-López, Víctor Manuel Navas-López, Francisco David Carmona, Teresa Nestares, Lara Bossini-Castillo

**Affiliations:** 1 Sección de Gastroenterología y Nutrición Infantil, Hospital Regional Universitario de Málaga, 29011 Málaga, Spain; 2 Instituto de Nutrición y Tecnología de los Alimentos “José Mataix Verdú” (INYTA), Centro de Investigación Biomédica (CIBM), Universidad de Granada, 18016 Granada, Spain; 3 Departamento de Genética e Instituto de Biotecnología, Centro de Investigación Biomédica (CIBM), Universidad de Granada, 18016 Granada, Spain; 4 Reproducción Humana y Enfermedades Hereditarias y Complejas (IBS-TEC14), Terapias Avanzadas y Tecnologías Biomédicas, Instituto de Investigación Biosanitaria de Granada (ibs.GRANADA), 18012 Granada, Spain; 5 Departamento de Fisiología, Facultad de Farmacia, Universidad de Granada, 18071 Granada, Spain

**Keywords:** celiac disease, T-cell lymphoma, polymorphisms, risk factor, Mendelian randomization

## Abstract

Celiac disease (CeD) is an immune-mediated disorder triggered by gluten ingestion that damages the small intestine. Although CeD has been associated with a higher risk for cancer, the role of CeD as a risk factor for specific malignancies, such as enteropathy-associated T-cell lymphoma (EATL), remains controversial. Using two-sample Mendelian randomization (2SMR) methods and the summarized results of large genome-wide association studies from public repositories, we addressed the causal relationship between CeD and eight different malignancies. Eleven non-HLA SNPs were selected as instrumental variables (IVs), and causality estimates were obtained using four 2SMR methods: random-effects inverse variance-weighted, weighted median estimation, MR-Egger regression, and MR pleiotropy residual sum and outlier (MR-PRESSO). We identified a significant causal relationship between CeD and mature T/NK cell lymphomas. Under a multivariate Mendelian randomization model, we observed that the causal effect of CeD was not dependent on other known lymphoma risk factors. We found that the most instrumental IV was located in the *TAGAP locus*, suggesting that aberrant T cell activation might be relevant in the T/NK cell malignization process. Our findings provide new insights into the connection between immune imbalance and the development of severe comorbidities, such as EATL, in patients with CeD.

## 1. Introduction

Celiac disease (CeD) is an immune-mediated enteropathy triggered by ingested gluten, a protein largely present in wheat, rye, and barley [1]. The prevalence of CeD follows a parallel distribution to the DQ2 and/or DQ8 human leukocyte antigen (HLA) class II haplotypes, reaching around 1.4% of the population and even higher when a disease screening system is implemented [2,3]. Additionally, the incidence of CeD is growing over time throughout the world [4].

After the host and microbiome enzymes digest gluten in the intestinal lumen, gluten digestion products, such as omega-5-gliadin, penetrate into the lamina propria and can directly stimulate the innate immune response (gluten-dependent allergy) or, after deamination by activated transglutaminase 2, stimulate the development of autoimmune diseases [1].

In autoimmune conditions, the innate and adaptive immune response cells initiate an inflammatory cascade in which lymphocytes have a central role [5]. In this proinflammatory environment, B cells produce autoantibodies and are involved in cytokine secretion or antigen presentation to T cells [6]. In fact, plasma cells have recently been identified as the main cell type presenting an immunodominant gluten epitope in patients with CeD [7]. On the other hand, gluten-reactive CD4+ T cells orchestrate a pro-inflammatory response and activate the previously mentioned autoreactive B cells [5]. Furthermore, the stress and inflammatory signals activate tissue-resident intraepithelial CD8+ cytotoxic T lymphocytes (IE-CTLs) in the intestine, which mediate the killing of epithelial cells and tissue destruction [5].

T cells are key in causing and perpetuating the damage of the intestinal mucosa, as has been shown in studies with gluten challenge, in which dramatic changes in the CD8 T lymphocyte population have been reported [5,8].

CeD includes inflammation, villous atrophy, and crypt hyperplasia in the small bowel and, if untreated, can lead to diarrhea, abdominal distention, abdominal pain, weight loss, fatigue, and malnutrition [1]. Nevertheless, CeD has emerged as a systemic disease since the immune system of patients with CeD has a characteristic proinflammatory profile and fails in the self-recognition process, which might cause extraintestinal manifestations (dermatitis, osteoporosis, epilepsy, etc.) and malignancies [9].

CeD has been associated with higher cancer predisposition, especially in the case of hematological, lymphoproliferative, hepatobiliary, and pancreatic cancers [10]. Small bowel carcinoma risk is increased in CeD patients, and CeD is sometimes diagnosed at the time of or subsequent to the carcinoma diagnosis [11,12]. Additionally, a well-known comorbidity of CeD is enteropathy-associated T cell lymphoma (EATL), which is a rare mature T cell non-Hodgkin lymphoma that can be either pre-existent to or concomitant with CeD [13]. Although CeD predominantly affects females [9], EATL is more common in men, and it normally arises after the age of 70 years old with a poor prognosis [10,14]. Moreover, EATL is often associated with refractory CeD type II, and the majority of EATL patients are homozygous for the HLA-DQ2 allele and had a late CeD diagnosis [15].

However, up to 90% of CeD patients carry HLA-DQ2.5 alleles (HLA-DQA1*05:01 and HLA-DQB1*02:01), while the rest express either HLA-DQ2.2 (HLA-DQA1*02:01 and HLA-DQB1*02:02; ~5%) or HLA-DQ8 (HLA-DQA1*03:01 and HLA-DQB1*03:02) alleles, and around 20% of the CeD patients are homozygous for HLA-DQ2 alleles [15,16]. These HLA alleles represent a clear genetic link with type I diabetes (T1D) and autoimmune thyroid disease, the most prevalent autoimmune disorders worldwide [17]. Despite this, only a small percentage of them develop RCDII (<1%) that progresses to EATL (30–50% of the RCDII patients) [9,13]. Moreover, genome-wide association studies (GWAS) have identified tens of non-HLA genetic polymorphisms associated with CeD [18,19], and CeD is known to have a complex inheritance pattern. Therefore, the disease onset and progression depend on a combination of genetic predisposition, gluten ingestion, and additional co-factors, such as stress, microbiota, diet, etc. [20]. Consequently, there is a specific interest in the field to better understand the possible causal relationship between CeD and cancers in general and EATL in particular.

Mendelian randomization (MR) methods might contribute to providing new insights into the role of CeD as a risk factor that triggers the development of different types of cancer. MR is based on the analysis of genetic variants, mostly single-nucleotide polymorphisms (SNPs), used as instrumental variables (IVs) to test whether a risk factor (exposure) has a causal effect on a disease (outcome) [21,22]. These methods rely on the fact that alleles are randomly distributed to the offspring and are present in the individuals before the onset of the disease. Therefore, the random inheritance of the alleles and their independence from the disease resemble the randomization during clinical trials and reduce the confounding effects of unknown factors [23]. Moreover, informative IVs should be associated with the exposure and not with confounding factors, and their effects should appear mediated exclusively by the risk factor [21,22]. Classical MR methods required that measurements of both the disease and the risk factor be obtained in the same set of individuals [21,22]. However, novel methods known as two-sample Mendelian randomization studies (2SMR) allow the use of the results of two independent studies: one study with measurements for the outcome and a different study with measurements for the risk factor, both sharing the same SNPs [24]. The IV information is often obtained from GWAS, which normally include the genotype information of thousands of individuals and hundreds of thousands of SNPs and have contributed to the identification of thousands of genetic risk factors for complex diseases [25]. Moreover, it is currently possible to implement 2SMR studies for multiple exposures and outcomes thanks to the availability of public repositories that offer free access to the results of GWAS carried out in large patient cohorts [26,27,28]. This strategy has been fruitful in cancer [29,30] and nutritional research [31], as well as in the study of cardiovascular [32,33], immune-mediated [34,35,36,37], and neurological disorders [38,39], amongst others.

Therefore, considering the advances in the field and the increasing interest of the clinical community in knowing more about the origin of disease comorbidities, we aimed to use avant-garde 2SMR methods to study the causal relationships between CeD and different cancers and malignancies in European populations.

## 2. Results

### 2.1. Eleven Non-HLA SNPs Associated with Celiac Disease Are Selected as IVs

We identified 11 non-HLA SNPs associated with CeD that reached the genome-wide significance level (*p*-value < 5 × 10^−8^) in the discovery cohort described by Dubois et al. [18]. The F-statistic reflected both a strong combined strength of the SNPs as IVs (F statistic > 500) and a strong effect of each individual IV separately (F statistic > 30) [40]. We extracted the summarized results of genetic association for these SNPs or their best available proxies from the GWAS of 8 traits related to “lymphoma” or “small bowel cancer”.

The minor alleles of 9 out of the 11 selected SNPs were associated with increased susceptibility to suffer from CeD (Table 1). However, we observed different allelic effects of these variants depending on the outcome (Table 1). Nevertheless, the genetic associations of the IVs with the outcomes were not significant at the genome-wide level of significance (Table 1). All the selected IVs were independent common variants in the human genome (MAF > 0.05) and did not represent palindromic changes (Table 1).

We analyzed previously reported genetic associations of the selected IVs with known lymphoma confounding factors, such as obesity-related traits [41,42] or smoking [43,44] (Supplementary Table S3). Only the variant rs653178 showed evidence of association with possible confounding factors for lymphoma (Supplementary Table S3).

### 2.2. Celiac Disease Is a Risk Factor for the Development of Mature T and NK Cell Lymphoma

**Figure 1:**
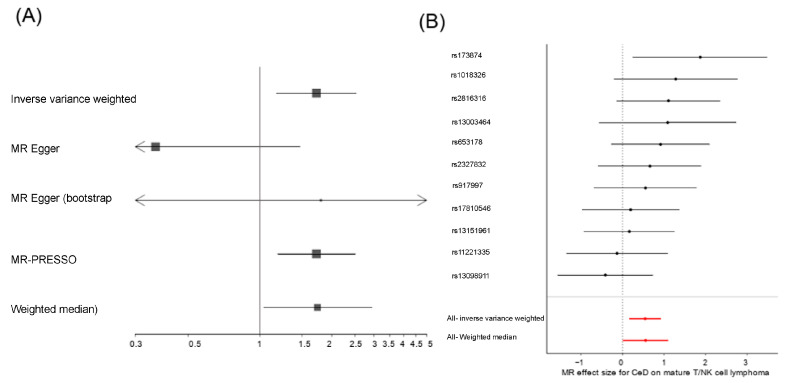
(**A**) Forest plot of the causal relationship effect of CeD and (**B**) Wald-ratio test of the effect of each CeD IV on mature T/NK cell lymphomas.

Four gold-standard methods of the 2SMR analysis were applied to the IV estimates to detect a causal relationship between CeD and relevant CeD-associated malignancies. Regarding the development of small bowel cancer, no significant risk effect of CeD was found (Table 2). When considering all types of lymphoma together as a unique outcome, no causal effect of CeD was revealed either (Table 2). However, when different lymphoma subsets were analyzed separately, we observed that CeD acted as a risk factor only for mature T/NK cell lymphomas (Figure 1, Supplementary Figure S1, Table S2). The causality relationship was supported by three different MR models: WM (*p*-value_WM_ = 4.12 × 10^−2^), IVW (*p*-value_IVW_ = 5.32 × 10^−3^), and MR-PRESSO (*p*-value_MR-PRESSO_ = 1.79 × 10^−2^). Horizontal pleiotropy analysis showed a marginal influence of a pleiotropic effect (*p*-value_Pleiotropy_ = 4.97 × 10^−2^). However, MR-PRESSO, which is especially focused on identifying and controlling for horizontal pleiotropy, did not identify any of the selected SNPs as outlier IVs and no heterogeneity was found in the IVW analysis (Q_IVW_ = 0.47).

It should be noted that the estimated effect was similar under the WM (OR_WM_ = 1.74, 95%CI [1.02–2.97]), the IVW (OR_IVW_ = 1.72, 95%CI [1.18–2.53]), and the MR-PRESSO (OR_MR-PRESSO_ = 1.72, 95%CI [1.18–2.52]) models. Additionally, the IVW and the MR-PRESSO models remained significant even after the removal of the possible confounder SNP rs653178 (*p*-value_IVW_ = 1.67 × 10^−2^, *p*-value_MR-PRESSO_ = 4.03 × 10^−2^, respectively).

The FinnGen repository harbors the GWAS results for different traits, and, in the case of malignancies, two analyses per trait were performed, i.e., an analysis including cancer patients in the control group (except for those affected by the specific cancer in question) and an analysis excluding any cancer patients from the control set. We analyzed the effect of CeD in all the lymphoma subsets included in FinnGen using the results of both analyses (including and excluding cancer patients from the control group) with similar results (Supplementary Tables S4 and S5).

We also discarded the possibility of a bidirectional causal effect between CeD and mature T/NK cell lymphoma by analyzing the effect of this malignancy as a risk factor for CeD. We considered twelve SNPs associated with mature T/NK cell lymphoma (*p*-value < 5 × 10^−5^), but we found no significant effect under any of the MR models (*p*-value_Egger_ = 1.88 × 10^−1^, *p*-value_WM_ = 3.34 × 10^−1^, *p*-value_IVW_ = 3.38 × 10^−1^, *p*-value_MR-PRESSO_ = 4.01 × 10^−1^).

### 2.3. Celiac Disease Is Independent of Other Mature T and NK Cell Lymphoma Risk Factors

As an additional step to control for possible weak effects of the SNPs as IVs for other risk factors, we repeated the 2SMR analyses after the removal of rs653178, which might be associated with risk factors for lymphoma (Supplementary Table S3). The risk effect MR models excluding this SNP remained significant under both the IVW (*p*-value_IVW_ = 1.67 × 10^−2^) and the MR-PRESSO (*p*-value_MR-PRESSO_ = 4.03 × 10^−2^) methods, but the WM method showed non-significant results (*p*-value_WM_ = 1.49 × 10^−1^).

Therefore, we decided to use a multivariable regression method (MVMR) to analyze the effect of CeD while controlling for the effects of other known lymphoma risk factors, such as obesity-related traits and smoking (Supplementary Table S6).

We decided to use past tobacco smoking (PTS) as a proxy for smoking effects since we observed an initial risk association of PTS with mature T/NK cell lymphoma in a univariate analysis under the MR Egger model (*p*-value_Egger_ = 2.93 × 10^−2^). However, amongst the most common obesity-related traits, only waist circumference (WC) showed suggestive risk associations with this type of lymphoma under both the WM model (*p*-value_WM_ = 7.97 × 10^−2^) and the IWV model (*p*-value_IVW_ = 6.64 × 10^−2^).

Consequently, we decided to generate a combined MVMR model including CeD, PTS, and WC as exposures for mature T/NK cell lymphoma. The MVMR analysis included a combined set of 91 independent IVs. We observed that the effects of the three risk factors remained significant in the combined model. Moreover, these effects did not depend on the IV selection, and they were significant either using all IVs from all exposures together or using only the exposure-specific IVs (Supplementary Table S6). However, the exposure-specific IV MVMR model allowed us to test the robustness of the CeD IVs, and we could observe that the estimated risk effect of CeD was not deeply affected by controlling for additional risk factors (Table 2 and Supplementary Table S6). The estimated effects of PTS and WC were greater (OR > 5) than the observed effect for CeD (OR > 1.4) (Supplementary Table S6). However, if the risk factors were analyzed in pairs, they showed independent effects. The only exception was the MVMR model including only CeD and PTS, in which the CeD risk effect was only suggestive (*p*-value_MVMR_ = 7.18 × 10^−2^) and the effect of PTS was not significant (*p*-value_MVMR_ = 3.20 × 10^−1^).

### 2.4. Celiac Disease-Associated T Cell Activation Is Linked with Mature T and NK Cell Lymphoma

We confirmed that the overall risk effect of CeD on mature T/NK cell lymphoma was not due to a single variant using a leave-one-out strategy (Supplementary Figure S2). However, using a Wald ratio test, we observed that the only IV that showed a significant causal relationship when considered separately, which considers the ratio between the variant-outcome association and the variant-exposure association [45], was the rs1738074 SNP (*p*-value_Wald-ratio_ = 2.33 × 10^−2^, OR = 6.55, 95% CI [1.29–32.77], Figure 1B).

The rs1738074 SNP is a biallelic (T > C) variant that is located in the 5′ UTR region of the *TAGAP locus* (near the transcription start site). *TAGAP* encodes a T cell activation RhoGTPase activating protein found in the cytosol of immune cells, especially in T cells and B cells [46] (Supplementary Figure S3). We could observe that the expression of this gene in the gut was also restricted to the T and B cells (Supplementary Figure S4) [46]. Considering the relevance of this locus in immune-mediated diseases (IMDs), we explored its possible functional implications using publicly available gene expression and epigenetic datasets.

The minor allele of the selected variant, namely rs1738074-T, increases the risk of suffering from both CeD and mature T/NK cell lymphoma (Table 1). The rs1738074-T allele showed an eQTL effect on *TAGAP* expression and led to decreased *TAGAP* expression in different tissues (Supplementary Table S7).

Additionally, the SNP was located in a highly transcriptionally active region classified as an active promoter in 12 tissues (including several subsets of T cells and B cells) and as an open chromatin region in 16 tissues, including the small intestine and B cells, T cells, and NK cells from peripheral blood [47]. Moreover, this polymorphism was predicted to be potentially damaging (PHRED > 10) by the CADD algorithm (PHRED_rs1738074_ = 12.150) [48]. Finally, chromatin immuno-precipitation sequencing (ChIP-seq) for several transcription factors identified the binding of NFKB (GEO accession: GSM935526, Supplementary Figure S5) and multiple proteins to this sequence.

## 3. Discussion

The link between CeD and malignancies, such as small bowel cancer and EATL, is well known [10,11,12,13]. In this study, we applied a novel 2SMR strategy to analyze the role of CeD as a risk factor for these malignancies based on the use of non-HLA CeD-associated genetic variants as IVs. By these means, we were able to provide novel insight into the connection between CeD and the pathogenesis of mature T/NK cell lymphoma.

Cancer is a complex disease with often unknown or uncertain risk factors [49]. Moreover, it is well known that there is a connection between IMDs and cancer [50]. In this context, 2SMR has provided the scientific community with tools to use IMD-associated SNPs as IVs to study the effect of IMDs on different cancers, based on the results of large and independent GWAS. For example, 2SMR strategies have identified (1) multiple sclerosis (MS) [51] and eosinophilia [52] as risk factors for lung cancer, (2) different levels of cytokines as risk or protective factors for multiple malignancies [53], and (3) systemic lupus erythematosus as a risk factor for lymphoma [54]. It is remarkable that the estimated risk effects of CeD on lymphoma in this study (OR > 1.7, Table 2) are in the range of the previously observed risk effects of proinflammatory cytokines on specific cancers [53], but considerably stronger than the effects of other IMDs [51,54]. We hypothesize that this strong effect might be due to the high correlation between CeD and EATL, which is a particular subtype of mature T-cell lymphoma, and the fact that we were able to study different subtypes of lymphoma separately. Therefore, the availability of summary statistics of mature T/NK cell lymphoma from the FinnGen repository GWAS was key to identifying a very specific risk for a certain lymphoma subtype, as we observed no significant effects neither on other subtypes of Hodgkin and non-Hodgkin lymphoma nor when considering all lymphomas together. These findings highlight the importance of generating subtype-specific GWAS data for complex diseases, especially if they are as heterogeneous as lymphoma. Nevertheless, the analyzed mature T/NK cell lymphoma cohort had a limited size (Supplementary Table S1), making statistical power one of the major caveats of this study. Moreover, the Finnish population has been found to have genetic ancestry differences from other European populations [55], which is a limitation of this study. Consequently, subsequent replication studies should be desirable to validate the reported results.

Using an MVMR model, we showed that the CeD risk effect on mature T/NK cell lymphoma is independent of other known lymphoma risk factors. Although the risk effects of smoking and obesity were greater than the effects of CeD, it should be noted that these differences will likely be affected by the removal of the CeD genetic markers in the HLA region (excluded to prevent statistical bias). Therefore, the estimated risk effect of CeD would be stronger if the HLA variants were considered. In any case, the independence between CeD and other risk factors opens the door to identifying lifestyle habits as additional environmental triggers that might have an accumulative risk effect on the onset and maintenance of EATL.

Finally, it should be noted that the over-representation of DQ2 homozygotes in EATL patients is clear, but not all the individuals who are homozygous for the HLA-DQ2 allele develop EATL (and nor all of them suffer from CeD) [15,16]. Therefore, additional CeD genetic susceptibility factors might be implicated in generating a pathological environment that might eventually favor the onset of EATL. Interestingly, we found that the most instrumental SNP in the mature T/NK cell lymphoma MR models corresponded to rs1738074, which maps in the vicinity of *TAGAP*, a *locus* that has been recently associated with hepatitis B virus-associated follicular lymphoma [56]. Remarkably, the minor allele of this genetic polymorphism, namely rs1738074-T, increases the risk of suffering from CeD [18,57], but it also increases the risk for Crohn’s disease (CD) [58,59], allergic disease, and asthma [60,61]. However, rs1738074-T has a protective effect on MS [62], rheumatoid arthritis (RA) [63,64], and T1D [65,66,67]. This apparent discrepancy in the association of *TAGAP* genetic variants with IMDs might be linked with its function in T cell activation, as observed in other master regulators of the immune response such as *PTPN22*. A missense variant in exon 14 of *PTPN22* (resulting in an arginine to tryptophan change at position 620 of the encoded protein) represents a strong genetic risk factor for some IMDs (e.g., RA, T1D, or systemic lupus erythematosus) and a protective variant for others (e.g., CD) [68]. In this sense, the protein encoded by *TAGAP* competes with ZAP70 for RhoH binding, thus reducing TCR signaling upon T cell activation [69]. Increased levels of *TAGAP* have been observed in the active states of multiple IMDs, for instance, CeD [70], RA [71], and CD [72], and this upregulation of *TAGAP* expression has been connected with an increased generation of Th17 cells that would create a pro-inflammatory environment and promote aberrant and chronic inflammation [73]. On the contrary, the risk allele for CeD, other gut or allergic IMDs, and EATL is associated with a lower expression of *TAGAP*. It might be possible that in specific body environments, such as the gut or the airways, that are constantly exposed to and interacting with microbes, products of digestion, or air pollutants, lower levels of TAGAP could support an increased TCR-driven activation and might contribute to the malignization of T cells in the long term. Furthermore, gene expression should not be considered an isolated event but rather a part of a highly interconnected metabolic network. Additional *loci* connected with *TAGAP*, such as *SH2B3*, have also been identified as genetic risk factors for CeD [18]. SH2B3 belongs to the SH2B adaptor family of proteins and has been associated with the involved development of CeD gastrointestinal and extraintestinal manifestations, CeD-associated conditions (autoimmune thyroid disease and T1D), and small bowel mucosal damage [74]. Interestingly, one of the IVs included in the present 2SMR study (rs653178) is a proxy for rs3184504, which is located in an exon of SH2B3 and acts as a trans-eQTL for *TAGAP* in peripheral blood [75]. Therefore, the dual role of *TAGAP* in different IMDs might be mediated by epistatic connections with other loci. The study of the possible role of the *TAGAP locus* in EATL might be a promising follow-up to our findings. Moreover, the links with environmental factors such as a gluten-free diet (GFD) should be further explored in the future, since the effect of a GFD in preventing the development of this tumor is not known [76]. Furthermore, elucidating this problem highlights the need for early diagnosis in asymptomatic subjects with the disease.

In conclusion, we have identified a consistent risk effect of CeD on mature T/NK cell lymphoma using non-HLA SNPs as IVs. This causal relationship between CeD and mature T/NK cell lymphoma is very specific to this lymphoma subtype and independent of other risk factors. Our results clearly suggest that aberrant T cell activation in CeD might contribute to an increased risk of developing mature T/NK cell lymphomas, such as EATL.

## 4. Materials and Methods

### 4.1. Genome-Wide Association Studies

**Figure 2:**
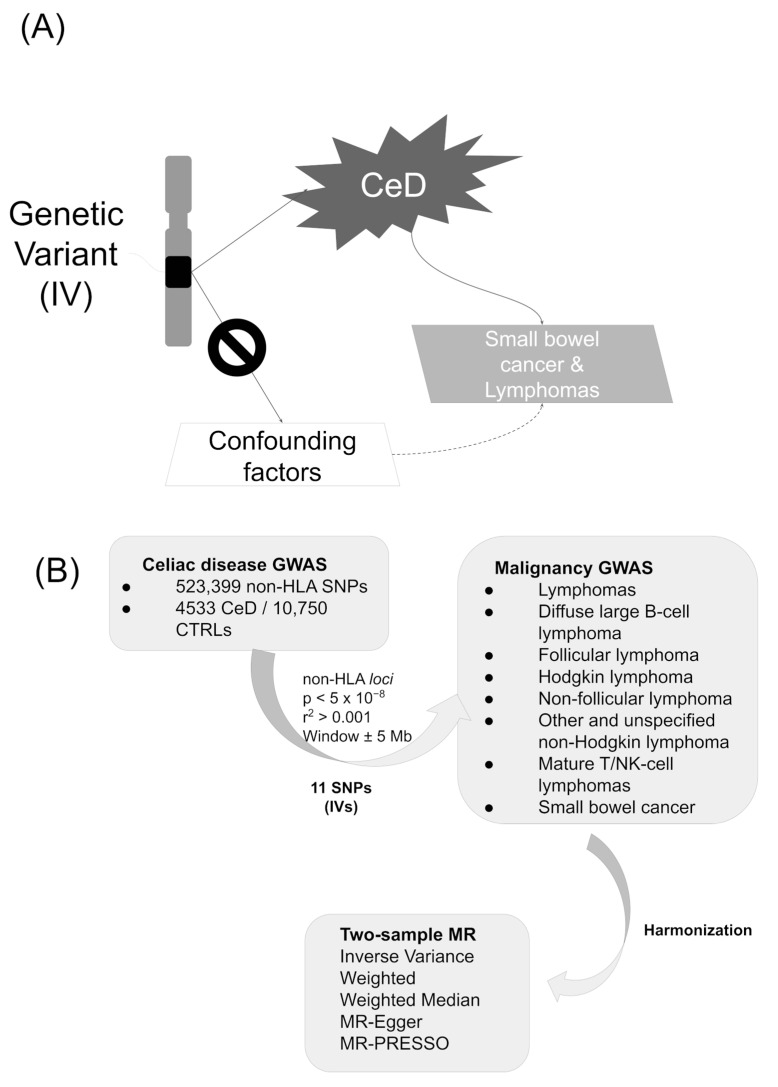
(**A**) Conceptual approach and (**B**) study design.

In this report, we considered CeD as the risk factor (exposure) and different malignancies as disease (outcomes), and we followed a 2SMR approach (Figure 2A).

The information for the CeD IVs was obtained from a previously published GWAS, in which 523,399 SNPs were analyzed in a discovery cohort including 4533 CeD patients and 10,750 healthy controls [18]. All the individuals had European ancestry, and the estimates were obtained in a meta-analysis of 5 different cohorts [18]. All the selected CeD IVs showed *p*-values < 5 × 10^−8^ in the discovery cohort of Dubois et al. [18] and were independent of each other (LD clumping parameters established at r2 > 0.001 in the 1000 Genomes European population [77] and distance ± 5 Mb) (Figure 2B).

We analyzed 8 different types of malignancies as outcomes (Supplementary Table S1). The summarized GWAS results of the outcomes were curated, quality controlled, and harmonized, being classified as any type of “lymphoma” (7 traits) or “small bowel cancer” (1 trait) in the IEU GWAS database [26,78] (Figure 2B). They were obtained from the cohorts included in the largest European biobanks: the UK Biobank (with genotype information for more than 10 million SNPs) and FinnGen (with genotype information for more than 16 million SNPs). The outcome definitions were defined elsewhere [77,79] and the selected traits included hundreds of cases and thousands of controls (Supplementary Table S1).

To the best of our knowledge, there were no overlapping individuals between the exposure and the outcome cohorts.

### 4.2. Mendelian Randomization Analysis

IVs located in the extended HLA region (chromosome 6: 20,000,000–40,000,000 bp) were excluded from the analyses to prevent bias due to the strong genetic association of the DQ2 and DQ8 alleles with CeD.

If the estimates for a specific IV were not present in the outcome data, the best available proxy was selected (according to the LD patterns observed in the European cohort of the 1000 Genomes Project). The 11 selected CeD IVs (or their statistically equivalent proxies) were present in all the outcome datasets (Table 1).

We applied gold-standard 2SMR methods as implemented in the “TwoSampleMR” [26] and the “MR-PRESSO” [80] R packages. We selected four well-established 2SMR methods to test for causality: (i) Random-effects inverse variance-weighted (IVW) MR, which calculates a combined causal effect as the sum of the weighted causal effects of all the SNPs (weighting is based on the inverse variance of the effect estimator) [21]; (ii) weighted median (WM) estimation, which can maintain consistency even if up to 50% of the IVs are invalid estimators and where a weighted median estimator is assigned to all the ratio estimates [81]; (iii) MR-Egger regression method, which can estimate the causal effect even if all the SNPs are weak or invalid IVs [22]; and (iv) MR pleiotropy residual sum and outlier (MR-PRESSO), which is able to find outlier IVs with horizontal pleiotropy and provide causality estimates after the removal of the outlier IVs [80].

False discovery rate (FDR) Benjamini and Hochberg method for multiple testing correction [82] was applied, and the models with pFDR < 0.05 were considered statistically significant.

We considered that the generated 2SMR models reached an appropriate statistical power to detect modest to relevant causal effects by using the MR study power calculations as implemented in Brion et al. [83] (Supplementary Table S2).

### 4.3. Sensitivity Analysis

All the significant models were validated after removing SNPs associated with known lymphoma confounding factors such as obesity [41,42], and tobacco smoking [43,44] (Supplementary Table S3), as reported by the PhenoScanner v2 [84].

For every exposure-outcome pair that showed a significant causality relation in at least two 2SMR models, the contribution of each SNP to the model was tested using a leave-one-out sensitivity analysis, as implemented in the “TwoSampleMR” package [26].

### 4.4. Multivariable Mendelian Randomization Analysis

As an additional check of the risk-effect reliability, we applied a multivariable Mendelian randomization (MVMR) [85] analysis as implemented in the TwoSampleMR package [26]. MVMR allowed us to estimate the effect of CeD while controlling for the effect of known risk factors for lymphoma that might act as confounding factors. For this analysis, we generated an IV dataset including the most associated SNPs with the 3 major anthropometric measurements from the GIANT consortium [86]. The GWAS results for body mass index (BMI), waist circumference (WC), and waist-to-hip ratio (WHR) were deposited in the IEU OpenGWAS repository under the following ids: ieu-a-2, ieu-a-67, and ieu-a-73, respectively. Nevertheless, only WC showed a marginal causal association with mature T/NK lymphoma and was included in the MVMR model. We also included past tobacco smoking (PTS, IEU OpenGWAS id: ukb-b-2134) in the MVMR as a proxy for tobacco exposure. After LD-clumping, 91 SNPs remained, and we generated a model that regressed CeD and the other risk factors together against the outcome and weighted for its inverse variance (in this case, mature T-NK cell lymphoma).

### 4.5. Functional Effect of the IVs

The functional implications of the relevant IVs were analyzed according to the information available in the most relevant public repositories focused on functional genomics, i.e., GTEx [87], SNPnexus [48], HaploReg [47], ENCODE [88,89], and the Human Protein Atlas [46].

## Figures and Tables

**Table 1:** IV genetic association results in the original GWAS datasets. Where celiac disease is the exposure and small bowel cancer or different lymphoma subtypes are the outcome.

Outcome	SNP	Chr	Position (bp)	Effect Allele	Other Allele	Beta Exposure	Pval Exposure	Beta Outcome	Pval Outcome	Eaf Outcome
Diffuse large B-cell lymphoma	rs1018326	2	182,007,800	C	T	0.1519	3.78 × 10^−9^	5.00 × 10^−4^	9.96 × 10^−1^	0.4994
	rs11221335	11	128,385,906	C	T	0.2175	4.16 × 10^−11^	−3.88 × 10^−2^	7.38 × 10^−1^	0.2342
	rs13003464	2	61,186,829	G	A	0.1415	4.92 × 10^−8^	−3.34 × 10^−2^	7.42 × 10^−1^	0.3696
	rs13098911	3	46,235,201	T	C	0.2784	2.53 × 10^−11^	9.46 × 10^−2^	4.98 × 10^−1^	0.1461
	rs13151961	4	123,115,502	G	A	−0.3239	6.31 × 10^−18^	1.99 × 10^−1^	1.95 × 10^−1^	0.1145
	rs1738074	6	159,465,977	C	T	−0.1424	3.14 × 10^−8^	−6.17 × 10^−2^	5.36 × 10^−1^	0.5824
	rs17810546	3	159,665,050	G	A	0.3235	4.56 × 10^−18^	−1.61 × 10^−1^	3.25 × 10^−1^	0.09909
	rs2327832 (rs6920220)	6	138,006,504	G	A	0.2319	1.41 × 10^−14^	−1.02 × 10^−1^	4.11 × 10^−1^	0.1888
	rs2816316	1	192,536,813	A	C	0.2544	1.45 × 10^−12^	2.00 × 10^−1^	1.47 × 10^−1^	0.8538
	rs653178	12	112,007,756	T	C	−0.1923	6.03 × 10^−14^	1.54 × 10^−1^	1.22 × 10^−1^	0.584
	rs917997	2	103,070,568	C	T	−0.2319	5.97 × 10^−15^	3.00 × 10^−2^	8.09 × 10^−1^	0.8084
Follicular lymphoma	rs1018326	2	182,007,800	C	T	0.1519	3.78 × 10^−9^	−3.07 × 10^−2^	6.22 × 10^−1^	0.4994
	rs11221335	11	128,385,906	C	T	0.2175	4.16 × 10^−11^	−1.26 × 10^−2^	8.64 × 10^−1^	0.2342
	rs13003464	2	61,186,829	G	A	0.1415	4.92 × 10^−8^	7.02 × 10^−2^	2.74 × 10^−1^	0.3696
	rs13098911	3	46,235,201	T	C	0.2784	2.53 × 10^−11^	−7.04 × 10^−2^	4.25 × 10^−1^	0.1461
	rs13151961	4	123,115,502	G	A	−0.3239	6.31 × 10^−18^	−1.03 × 10^−1^	2.93 × 10^−1^	0.1145
	rs1738074	6	159,465,977	C	T	−0.1424	3.14 × 10^−8^	−1.77× 10^−2^	7.79 × 10^−1^	0.5824
	rs17810546	3	159,665,050	G	A	0.3235	4.56 × 10^−18^	−1.01 × 10^−1^	3.30 × 10^−1^	0.09909
	rs2327832 (rs6920220)	6	138,006,504	G	A	0.2319	1.41 × 10^−14^	−7.97 × 10^−2^	3.13 × 10^−1^	0.1888
	rs2816316	1	192,536,813	A	C	0.2544	1.45 × 10^−12^	−5.29 × 10^−2^	5.46 × 10^−1^	0.8538
	rs653178	12	112,007,756	T	C	−0.1923	6.03 × 10^−14^	−6.25 × 10^−2^	3.21 × 10^−1^	0.584
	rs917997	2	103,070,568	C	T	−0.2319	5.97 × 10^−15^	5.11 × 10^−2^	5.15 × 10^−1^	0.8084
Hodgkin lymphoma	rs1018326	2	182,007,800	C	T	0.1519	3.78 × 10^−9^	8.30 × 10^−3^	9.11 × 10^−1^	0.4994
	rs11221335	11	128,385,906	C	T	0.2175	4.16 × 10^−11^	7.96 × 10^−2^	3.61 × 10^−1^	0.2342
	rs13003464	2	61,186,829	G	A	0.1415	4.92 × 10^−8^	1.83 × 10^−1^	1.70 × 10^−2^	0.3696
	rs13098911	3	46,235,201	T	C	0.2784	2.53 × 10^−11^	−1.14 × 10^−1^	2.81 × 10^−1^	0.1461
	rs13151961	4	123,115,502	G	A	−0.3239	6.31 × 10^−18^	−7.82 × 10^−2^	5.01 × 10^−1^	0.1145
	rs1738074	6	159,465,977	C	T	−0.1424	3.14 × 10^−8^	2.11 × 10^−2^	7.79 × 10^−1^	0.5824
	rs17810546	3	159,665,050	G	A	0.3235	4.56 × 10^−18^	4.27 × 10^−2^	7.30 × 10^−1^	0.09909
	rs2327832 (rs6920220)	6	138,006,504	G	A	0.2319	1.41 × 10^−14^	−2.15 × 10^−2^	8.19 × 10^−1^	0.1888
	rs2816316	1	192,536,813	A	C	0.2544	1.45 × 10^−12^	−7.50 × 10^−3^	9.43 × 10^−1^	0.8538
	rs653178	12	112,007,756	T	C	−0.1923	6.03 × 10^−14^	4.56 × 10^−2^	5.44 × 10^−1^	0.584
	rs917997	2	103,070,568	C	T	−0.2319	5.97 × 10^−15^	−6.82 × 10^−2^	4.68 × 10^−1^	0.8084
Non-follicular lymphoma	rs1018326	2	182,007,800	C	T	0.1519	3.78 × 10^−9^	−3.43 × 10^−2^	3.92 × 10^−1^	0.4994
	rs11221335	11	128385906	C	T	0.2175	4.16 × 10^−11^	4.88 × 10^−2^	3.00 × 10^−1^	0.2342
	rs13003464	2	61,186,829	G	A	0.1415	4.92 × 10^−8^	5.75 × 10^−2^	1.64 × 10^−1^	0.3696
	rs13098911	3	46,235,201	T	C	0.2784	2.53 × 10^−11^	−2.86 × 10^−2^	6.15 × 10^−1^	0.1461
	rs13151961	4	123,115,502	G	A	−0.3239	6.31 × 10^−18^	−1.06 × 10^−2^	8.66 × 10^−1^	0.1145
	rs1738074	6	159,465,977	C	T	−0.1424	3.14 × 10^−8^	−1.90 × 10^−2^	6.40 × 10^−1^	0.5824
	rs17810546	3	159,665,050	G	A	0.3235	4.56 × 10^−18^	6.50 × 10^−3^	9.22 × 10^−1^	0.09909
	rs2327832 (rs6920220)	6	138,006,504	G	A	0.2319	1.41 × 10^−14^	−7.19 × 10^−2^	1.57 × 10^−1^	0.1888
	rs2816316	1	192,536,813	A	C	0.2544	1.45 × 10^−12^	6.84 × 10^−2^	2.25 × 10^−1^	0.8538
	rs653178	12	112,007,756	T	C	−0.1923	6.03 × 10^−14^	9.04 × 10^−2^	2.58 × 10^−1^	0.584
	rs917997	2	103,070,568	C	T	−0.2319	5.97 × 10^−15^	5.16 × 10^−2^	3.09 × 10^−1^	0.8084
Other and unspecified types of non-Hodgkin lymphoma	rs1018326	2	182,007,800	C	T	0.1519	3.78 × 10^−9^	2.00 × 10^−3^	9.74 × 10^−1^	0.4994
	rs11221335	11	128,385,906	C	T	0.2175	4.16 × 10^−11^	7.59 × 10^−2^	2.97 × 10^−1^	0.2342
	rs13003464	2	61,186,829	G	A	0.1415	4.92 × 10^−8^	2.51 × 10^−2^	6.93 × 10^−1^	0.3696
	rs13098911	3	46,235,201	T	C	0.2784	2.53 × 10^−11^	−3.00 × 10^−2^	7.33 × 10^−1^	0.1461
	rs13151961	4	123,115,502	G	A	−0.3239	6.31 × 10^−18^	2.40 × 10^−3^	9.80 × 10^−1^	0.1145
	rs1738074	6	159,465,977	C	T	−0.1424	3.14 × 10^−8^	−1.11 × 10^−2^	8.60 × 10^−1^	0.5824
	rs17810546	3	159,665,050	G	A	0.3235	4.56 × 10^−18^	−7.88 × 10^−2^	4.43 × 10^−1^	0.09909
	rs2327832 (rs6920220)	6	138,006,504	G	A	0.2319	1.41 × 10^−14^	−6.06 × 10^−2^	4.39 × 10^−1^	0.1888
	rs2816316	1	192,536,813	A	C	0.2544	1.45 × 10^−12^	2.51 × 10^−2^	7.72 × 10^−1^	0.8538
	rs653178	12	112,007,756	T	C	−0.1923	6.03 × 10^−14^	5.10 × 10^−2^	4.14 × 10^−1^	0.584
	rs917997	2	103,070,568	C	T	−0.2319	5.97 × 10^−15^	−2.91 × 10^−2^	7.09 × 10^−1^	0.8084
Mature T/NK-cell lymphomas	rs1018326	2	182,007,800	C	T	0.1519	3.78 × 10^−9^	1.95 × 10^−1^	9.25 × 10^−2^	0.4994
	rs11221335	11	128,385,906	C	T	0.2175	4.16 × 10^−11^	−2.89 × 10^−2^	8.32 × 10^−1^	0.2342
	rs13003464	2	61,186,829	G	A	0.1415	4.92 × 10^−8^	1.54 × 10^−1^	1.97 × 10^−1^	0.3696
	rs13098911	3	46,235,201	T	C	0.2784	2.53 × 10^−11^	−1.15 × 10^−1^	4.86 × 10^−1^	0.1461
	rs13151961	4	123,115,502	G	A	−0.3239	6.31 × 10^−18^	−5.31 × 10^−2^	7.69 × 10^−1^	0.1145
	rs1738074	6	159,465,977	C	T	−0.1424	3.14 × 10^−8^	−2.67 × 10^−1^	2.33 × 10^−2^	0.5824
	rs17810546	3	159,665,050	G	A	0.3235	4.56 × 10^−18^	6.38 × 10^−2^	7.40 × 10^−1^	0.09909
	rs2327832 (rs6920220)	6	138,006,504	G	A	0.2319	1.41 × 10^−14^	1.53 × 10^−1^	2.97 × 10^−1^	0.1888
	rs2816316	1	192,536,813	A	C	0.2544	1.45 × 10^−12^	2.82 × 10^−1^	8.31 × 10^−2^	0.8538
	rs653178	12	112,007,756	T	C	−0.1923	6.03 × 10^−14^	−1.76 × 10^−1^	1.33 × 10^−1^	0.584
	rs917997	2	103,070,568	C	T	−0.2319	5.97 × 10^−15^	−1.28 × 10^−1^	3.82 × 10^−1^	0.8084
Lymphomas	rs1018326	2	182007800	C	T	0.1519	3.78 × 10^−9^	−5.26 × 10^−5^	7.51 × 10^−1^	0.42235
	rs11221335	11	128,385,906	C	T	0.2175	4.16 × 10^−11^	2.47 × 10^−4^	2.17 × 10^−1^	0.21227
	rs13003464	2	61,186,829	G	A	0.1415	4.92 × 10^−8^	3.00 × 10^−4^	7.58 × 10^−2^	0.37623
	rs13098911	3	46,235,201	T	C	0.2784	2.53 × 10^−11^	−1.79 × 10^−4^	5.64 × 10^−1^	0.07501
	rs13151961	4	123,115,502	G	A	−0.3239	6.31 × 10^−18^	8.67 × 10^−5^	6.87 × 10^−1^	0.17478
	rs1738074	6	159,465,977	C	T	−0.1424	3.14 × 10^−8^	9.34 × 10^−5^	5.70 × 10^−1^	0.56446
	rs17810546	3	159,665,050	G	A	0.3235	4.56 × 10^−18^	1.99 × 10^−4^	4.22 × 10^−1^	0.12335
	rs2327832	6	137,973,068	G	A	0.2319	1.41 × 10^−14^	6.79 × 10^−5^	7.30 × 10^−1^	0.22264
	rs2816316	1	192,536,813	A	C	0.2544	1.45 × 10^−12^	−2.90 × 10^−6^	9.89 × 10^−1^	0.81835
	rs653178	12	112,007,756	T	C	−0.1923	6.03 × 10^−14^	1.95 × 10^−4^	2.34 × 10^−1^	0.51724
	rs917997	2	103,070,568	C	T	−0.2319	5.97 × 10^−15^	−1.54 × 10^−4^	4.33 × 10^−1^	0.77549
Small intestine/small bowel cancer	rs1018326	2	182,007,800	C	T	0.1519	3.78 × 10^−9^	−7.65 × 10^−5^	1.49 × 10^−1^	0.42222
	rs11221335	11	128,385,906	C	T	0.2175	4.16 × 10^−11^	2.71 × 10^−5^	6.72 × 10^−1^	0.21196
	rs13003464	2	61,186,829	G	A	0.1415	4.92 × 10^−8^	−8.21 × 10^−5^	1.29 × 10^−1^	0.37657
	rs13098911	3	46,235,201	T	C	0.2784	2.53 × 10^−11^	1.15 × 10^−4^	2.47 × 10^−1^	0.07483
	rs13151961	4	123,115,502	G	A	−0.3239	6.31 × 10^−18^	2.26 × 10^−5^	7.43 × 10^−1^	0.17515
	rs1738074	6	159,465,977	C	T	−0.1424	3.14 × 10^−8^	−5.30 × 10^−5^	3.15 × 10^−1^	0.56542
	rs17810546	3	159,665,050	G	A	0.3235	4.56 × 10^−18^	1.73 × 10^−4^	2.96 × 10^−2^	0.12339
	rs2327832	6	137,973,068	G	A	0.2319	1.41 × 10^−14^	−1.87 × 10^−5^	7.66 × 10^−1^	0.22287
	rs2816316	1	192,536,813	A	C	0.2544	1.45 × 10^−12^	−1.32 × 10^−5^	8.47 × 10^−1^	0.81853
	rs653178	12	112,007,756	T	C	−0.1923	6.03 × 10^−14^	7.96 × 10^−5^	1.29 × 10^−1^	0.51709
	rs917997	2	103,070,568	C	T	−0.2319	5.97 × 10^−15^	−6.43 × 10^−5^	3.06 × 10^−1^	0.77487

**Table 2:** 2SMR results under different methods where celiac disease is the exposure and different lymphoma subtypes are the outcome 10×^−^.

Outcome	Method	Pval	OR	OR _l_CI95	OR _u_CI95
Lymphomas	MR-Egger	9.70 × 10^−1^	1.00	1.00	1.00
	Weighted median	9.68 × 10^−1^	1.00	1.00	1.00
	Inverse variance weighted	6.12 × 10^−1^	1.00	1.00	1.00
	MR-PRESSO	5.86 × 10^−1^	1.00	1.00	1.00
Diffuse large B-cell lymphoma	MR-Egger	5.34 × 10^−1^	0.68	0.21	2.21
	Weighted median	3.45 × 10^−1^	0.82	0.54	1.24
	Inverse variance weighted	3.34 × 10^−1^	0.85	0.62	1.18
	MR-PRESSO	3.06 × 10^−1^	0.85	0.64	1.14
Follicular lymphoma	MR-Egger	3.52 × 10^−1^	0.69	0.32	1.46
	Weighted median	1.42 × 10^−1^	0.81	0.62	1.07
	Inverse variance weighted	6.49 × 10^−1^	0.95	0.78	1.17
	MR-PRESSO	5.93 × 10^−1^	0.95	0.80	1.13
Hodgkin lymphoma	MR-Egger	5.82 × 10^−1^	0.77	0.31	1.89
	Weighted median	7.15 × 10^−1^	1.06	0.77	1.46
	Inverse variance weighted	4.66 × 10^−1^	1.10	0.86	1.40
	MR-PRESSO	4.55 × 10^−1^	1.10	0.87	1.38
Non-follicular lymphoma	MR-Egger	8.30 × 10^−1^	0.94	0.52	1.69
	Weighted median	9.70 × 10^−1^	1.00	0.82	1.20
	Inverse variance weighted	5.80 × 10^−1^	0.96	0.82	1.12
	MR-PRESSO	5.92 × 10^−1^	0.96	0.82	1.12
Other and unspecified types of non-Hodgkin lymphoma	MR-Egger	6.02 × 10^−1^	0.81	0.39	1.71
	Weighted median	9.87 × 10^−1^	1.00	0.77	1.30
	Inverse variance weighted	8.39 × 10^−1^	0.98	0.80	1.20
	MR-PRESSO	7.36 × 10^−1^	0.98	0.87	1.10
Mature T/NK-cell lymphomas	MR-Egger	1.91 × 10^−1^	0.36	0.09	1.48
	Weighted median	**4.12 × 10^−2^**	1.74	1.02	2.97
	Inverse variance weighted	**5.32 × 10^−3^**	1.72	1.18	2.53
	MR-PRESSO	**1.79 × 10^−2^**	1.72	1.18	2.52
Small intestine/small bowel cancer	MR-Egger	1.21 × 10^−1^	1.00	1.00	1.00
	Weighted median	6.46 × 10^−1^	1.00	1.00	1.00
	Inverse variance weighted	8.35 × 10^−1^	1.00	1.00	1.00
	MR-PRESSO	8.39 × 10^−1^	1.00	1.00	1.00

## Data Availability

All the summarized genetic association results of genome-wide association studies included in this manuscript were previously deposited in public repositories and they were accessible and free to download.

## Supplementary Materials

The following supporting information can be downloaded at: https://www.mdpi.com/article/10.3390/ijms24087216/s1.
