# Supplementary material for: Celiac Disease Is a Risk Factor for Mature T and NK Cell Lymphoma: A Mendelian Randomization Study
Source: Int J Mol Sci. 2023 Apr 13;24(8):7216. doi: 10.3390/ijms24087216 (PMC10139431; doi:10.3390/ijms24087216)

Supplementary Figure S3.

GENERAL INFORMATION	
**Gene name**	TAGAP
**Gene description**	T cell activation RhoGTPase activating protein
**Predicted location**	Intracellular
**Number of transcripts**	3

HUMAN PROTEIN ATLAS INFORMATION	
**Single cell type expression cluster**	Non-specific - Transcription (mainly)
**Single cell type specificity**	Cell type enhanced (Plasma cells, B-cells, T-cells, dendritic cells, Macrophages, NK-cells)
**Immune cell specificity**	Low immune cell specificity
**Immune cell distribution**	Detected in all
**Cell line specificity**	Cell line enhanced (Daudi, HMC-1, Karpas-707, U-266/84, U-698, U-937)
**Cell line distribution**	Detected in some
**Protein evidence**	Evidence at protein level

SINGLE CELL TYPES

Single cell types

RNA single cell type specificity: Cell type enhanced (Plasma cells, B-cells, T-cells, dendritic cells, Macrophages, NK-cells)

Group Expression Alphabetical

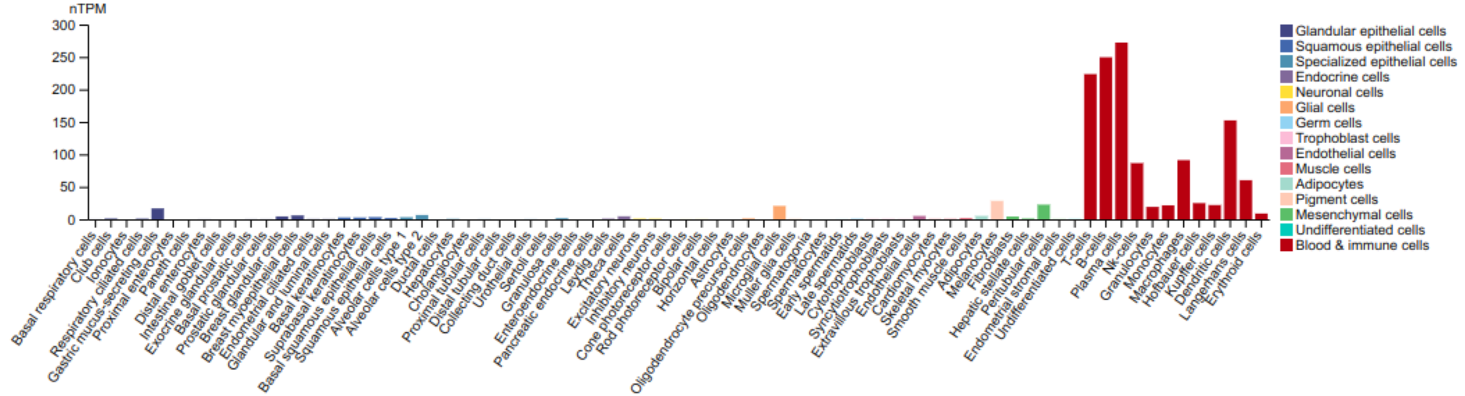

Supplement: Supplementary file 1 [file ijms-24-07216-s001.zip › Sup_Figure_S3.pdf]
