# Supplementary figures and images for: Celiac Disease Is a Risk Factor for Mature T and NK Cell Lymphoma: A Mendelian Randomization Study
Source: Int J Mol Sci. 2023 Apr 13;24(8):7216. doi: 10.3390/ijms24087216 (PMC10139431; doi:10.3390/ijms24087216)

Supplementary Figure S1.

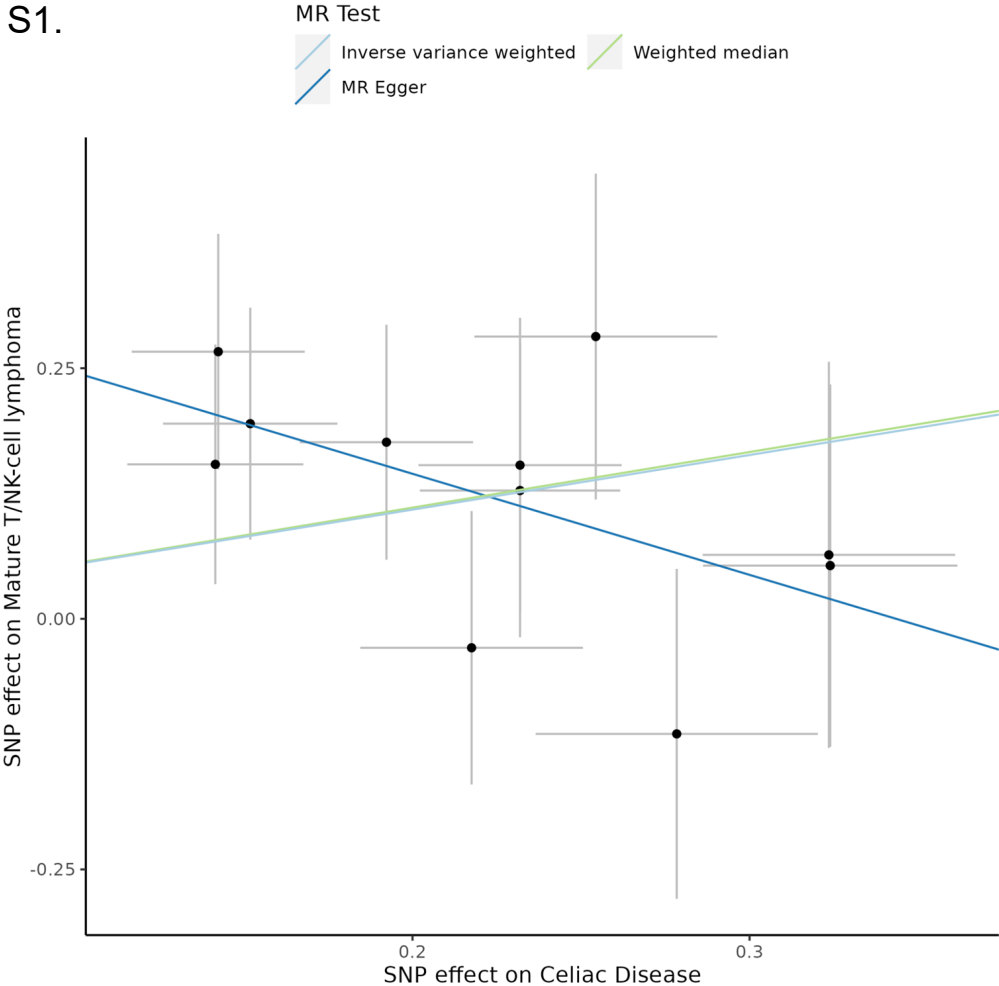

Supplement: Supplementary file 1 [file ijms-24-07216-s001.zip › Sup_Figure_S1.pdf]

Supplementary Figure S2.

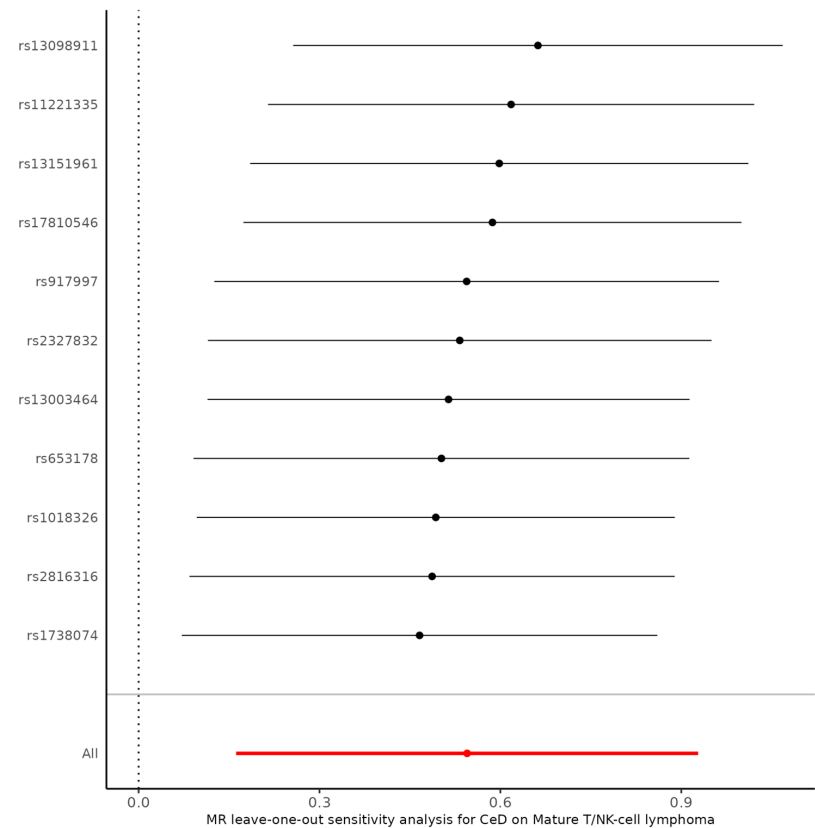

Supplement: Supplementary file 1 [file ijms-24-07216-s001.zip › Sup_Figure_S2.pdf]

Supplementary Figure S4.

A)

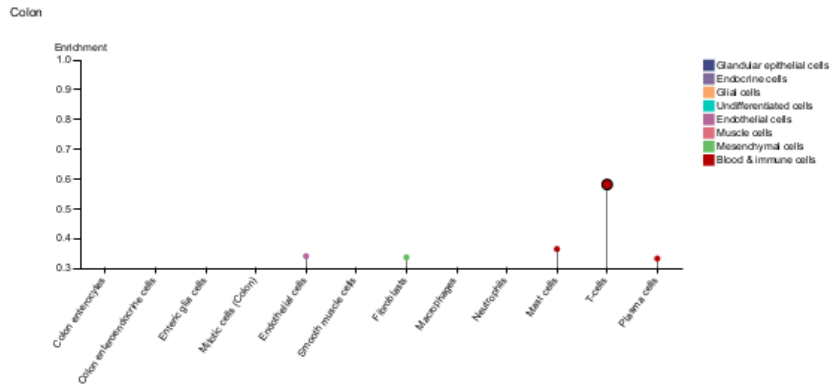

B)

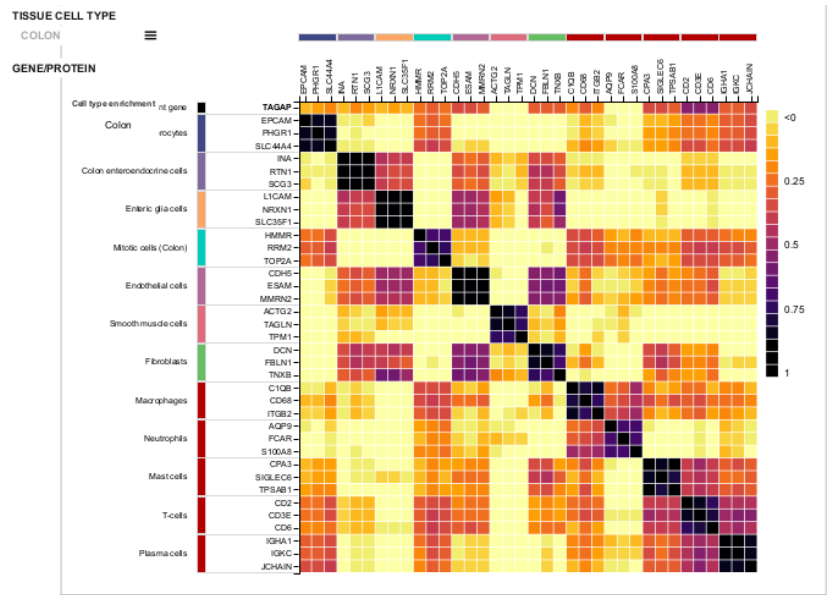

Supplement: Supplementary file 1 [file ijms-24-07216-s001.zip › Sup_Figure_S4.pdf]

Supplementary Figure S5.

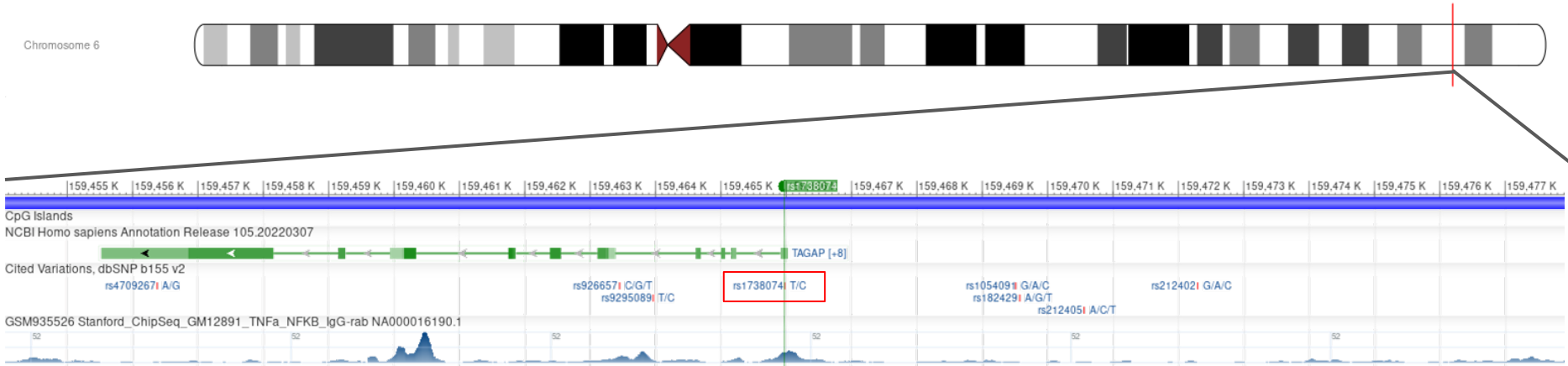

Supplement: Supplementary file 1 [file ijms-24-07216-s001.zip › Sup_Figure_S5.pdf]
